# Emotional Contagion in the Online Depression Community

**DOI:** 10.3390/healthcare9121609

**Published:** 2021-11-23

**Authors:** Jingyun Tang, Guang Yu, Xiaoxu Yao

**Affiliations:** School of Management, Harbin Institute of Technology, 92 Xidazhi Street, Nangang District, Harbin 150001, China; tang_jing_yun@126.com (J.T.); xiaoxu.yao06@gmail.com (X.Y.)

**Keywords:** emotional contagion, depression, online depression community, social media, text-mining

## Abstract

Negative emotions are prevalent in the online depression community (ODC), which potentially puts members at risk, according to the theory of emotional contagion. However, emotional contagion in the ODC has not been confirmed. The generalized estimating equation (GEE) was used to verify the extent of emotional contagion using data from 1548 sample users in China’s popular ODC. During interaction, the emotional themes were analyzed according to language use. The diurnal patterns of the interaction behaviors were also analyzed. We identified the susceptible groups and analyzed their characteristics. The results confirmed the occurrence of emotional contagion in ODC, that is, the extent to which the user’s emotion was affected by the received emotion. Our study also found that when positive emotional contagion occurred, the replies contained more hopefulness, and when negative emotional contagion occurred, the replies contained more hopelessness and fear. Second, positive emotions were easier to spread, and people with higher activity in ODC were more susceptible. In addition, nighttime was an active period for user interaction. The results can help community managers and support groups take measures to promote the spread of positive emotions and reduce the spread of negative emotions.

## 1. Introduction

Human emotions are strongly influenced by social interactions. Previous studies have shown that happiness [1], depression [2], loneliness [3], and other emotions can spread in social networks. Emotional contagion [4] is initially considered to occur between people who are often in close contact, such as families [5] and roommates [6,7]. Emotions also spread in face-to-face communication, such as during workplace interactions [6,8,9], and even in experimental situations [10].

The emergence of social media platforms has changed the traditional ways of communication. Personal emotions are expressed in texts that are posted online [11]. Various recent contributions have advanced the hypothesis that emotions may be spread throughout online social networks [12,13,14,15,16,17,18,19,20,21]. For example, a study found that after users update their status with emotional content, their friends are significantly more likely to make a valence-consistent post [22]. However, this study did not distinguish between emotional contagion and homogeneity; that is, users like to make friends and communicate with people with the same emotional state. To solve this problem, Kramer et al. [23] designed an experiment by reducing the amount of emotional content (positive and negative) in the news feed on Facebook and found that people produced fewer positive posts and more negative posts when positive expressions were reduced and vice versa. However, manipulation of the information that users see raises ethical concerns. In an empirical study [24], members of mental health communities were shown to experience significant increases in anxiety, anger, and negative emotions following reports of several celebrity suicides. In another study, it was shown that rainfall directly influenced the emotional content of Facebook users’ status messages and also affected the status messages of friends in other cities who were not experiencing rainfall [14]. In short, most of the research on emotional contagion based on social media is realized by observing the emotional expression of users after they see a status update with emotional content. Consequently, some studies compared the spread of different emotions and found some evidence that on social media, people usually share positive emotions more often than negative ones. However, anger is an exception [17]. Some studies show that posts containing anger are more likely to spread among several users [25].

The functioning of social media platforms may even intensify the negative aspects of outrage, such as harassment or potentially anger, instead of turning outrage into a force of social progress [26]. An online community, as a form of social media, helps users establish social networks, enhancing communication and mutual influence [27]. In recent years, an increasing number of online health communities have formed where people with the same diseases gather. Online health communities can increase opportunities for patients to communicate with their peers [28], reduce the stigma of illness [29], and provide social support that is not limited by space and time [30]. Therefore, it is generally believed that online health communities have a positive impact on users [31,32,33,34,35]. Research on the benefits of online health communities highlights psychosocial benefits, such as reduced depression [33,35], anxiety [33,36], stress [33], and negative emotions [37,38]. While these studies reveal the emotional changes of users, they do not consider the relationship between emotional changes and interaction. Furthermore, most of these studies are not based on mental health communities, such as the online depression community (ODC).

The prevalence of depression has been increasing every year of late. According to a WHO report, depression affects more than 350 million people worldwide and is the leading cause of disability [39]. Depressed individuals are reluctant to share their emotional states with family and friends, but they are willing to share with their peers in ODCs, as they may feel it is less risky to disclose their experiences online [40,41]. Negative emotion is a primary characteristic of depressive individuals and is also common in ODCs [42]. The emotional contagion theory [43] suggests that prolonged interactions with depressed individuals and their negative emotions can worsen the symptoms of depression [38]. In ODCs, users frequently communicate with numerous other users [14]. However, emotional contagion in interactions within ODCs has not been confirmed.

In the online community, users engage in text-based interactions, so when we study emotional contagion in an online community, we should consider whether the emotional content of communication among users will affect the emotion of receiving users. Considering the large amount of negative emotional content and emotional interaction in ODCs, it is necessary to study emotional contagion in this community. This study aims to verify the emotional contagion in ODC interactions. Based on the epidemic model, three factors of the emotional transmission process are analyzed: themes of emotional interaction, diurnal pattern of interaction, and characteristics of susceptible groups. The results provide suggestions for the management of ODCs to provide better support for community users.

## 2. Methods

### 2.1. Data Collection

In this study, the ODC under consideration comprises a comment thread appearing on Sina Weibo, which is one of the most popular social media platforms in China and is similar to Twitter [44]. In 2012, a Sina Weibo user “zoufan” died by suicide related to depression and posted a farewell posting. An increasing number of people suffering from depression or depression symptoms tend to post comments under the farewell posting to disclose their depression, express their feelings, and communicate with others. At present, over 1 million comments have been posted under the farewell posting, and the number continues to grow. Many studies have used this comment thread as an ODC to study depression-related issues, because it has the largest concentration of people with depressive symptoms and has more posts than other ODCs [45,46,47]. Moreover, as there is no speech restriction in this ODC, members can express their feelings and thoughts freely, even including thoughts on suicide. As a result, the community has numerous emotional posts, including negative emotions. Therefore, it provides a suitable data source for the study of emotional contagion. We developed a Python program to automatically download postings.

These postings are published anonymously on an open and accessible platform. Therefore, according to the ethical guidelines from Benton et al. [48], an ethical review was not required in this study. In addition, to protect users’ anonymity, any information that could be trace to users is hidden.

### 2.2. Data Selection

In order to verify the emotional contagion in ODC, we must first get the member’s longitudinal emotional changes, which requires that the member must stay in the community for a certain period of time. Those users who only stay in the community for a short period were not considered. Second, as emotional contagion is based on interaction, those members who do not interact with others in the community were not included in this study. We identified 1548 sample users for our study who were active in the community for 4 weeks or more and interacted with others at least once. Their original posts (*n* = 242,788) and interactive posts (*n* = 67,987) with others were extracted. In addition, their gender was retrieved. 

### 2.3. Emotional Analysis

We built a text classifier to identify emotions in postings. Classification is modeled as a supervised learning process in which a training dataset is required. The coding process for the training dataset was done as follows. First, two psychology students coded the 10,000 postings (randomly selected from the 242,788 original postings and 67,987 interactive posting) as positive, neutral, or negative. Second, where there is a disagreement, they discussed each of the postings until an agreement was reached. Finally, a third researcher who is also experienced in mental health coded 1000 postings randomly sampled from the 10,000 postings to compute the inter-rater reliability. All Kappas are >0.85.

We trained a text classifier using bidirectional encoder representations from transformers (BERT), which is a new language representation model developed by Google in 2018 that obtains new state-of-the-art results on 11 natural language processing tasks [49]. The accuracy of the trained classifier is 84.28%, and the F1 score is 83.54, which shows that the classifier has a good performance.

Each posting after classification has an emotional label. To obtain the user’s emotional state in a time period, we need to aggregate user postings to calculate the emotional value. The emotional value $EV_{it}$ for user *i* in the epoch *t* is defined as follows:

$$EV_{it}=\frac{TP_{it}-TN_{it}}{T_{it}}\;$$

where *TP_it_* is the total number of positive postings published by user *i* in the epoch *t*. *TN_it_* is the total number of negative posts published by user *i* in the epoch *t*. *T_it_* is the total number of posts published by user *i* in the epoch *t*. The closer a user’s $EV_{it}$ is to 1, the more positive his or her emotion will be. On the contrary, the closer $EV_{it}$ is to −1, the more negative the user’s emotion is.

The users’ received emotional value $REV_{it}$ from the received replies is calculated as follows:

$$REV_{it}=\frac{TRP_{it}-TRN_{it}}{TR_{it}}\;$$

where *TRP_it_* is the total number of received positive replies by user *i* in the epoch *t*. *TRN_it_* is the total number of received negative replies by user *i* in the epoch *t*. *TR_it_* is the total number of all replies received by user *i* in the epoch *t*. The closer a user’s $REV_{it}$ value is to 1, the more positive the emotion the user receives. On the contrary, the closer a user’s $REV_{it}$ value is to −1, the more negative the emotion the user receives.

### 2.4. Longitudinal Changes

First, we wanted to understand the emotional change of these users during their participation in the community. Therefore, we calculated and clustered the user’s longitudinal emotional changes. Specifically, we divided the total time of user’s active engagement in the community into three equal time spans: the early, middle, and late periods of participation. Then, the values of *EV_it_* in each period were calculated according to the method mentioned in Section 2.3. Thus, we obtained the 1 × 3 vector of each user’s longitudinal emotion change. Finally, we applied the classic *k*-means clustering algorithm to cluster all the user’s longitudinal emotional changes. The optimal number of clusters from the *k*-means clustering results was estimated using the R package NbClust [50].

### 2.5. Statistical Analysis of Emotional Contagion

After determining the emotional changes of users during their participation in the community, we wanted to know whether these changes were affected by the interaction with others in the community, that is, whether there is an emotional contagion. We observed the emotional changes of users on a weekly basis and investigated whether their emotions in the current week are affected by the emotions received in the current week. In addition, considering the lag of the impact, the emotions received in the previous week may also affect the users’ emotional expressions in the current week. Thus, we hypothesize the following:

**Hypothesis** **1** **(H1).** *The emotions received by users in the current week have a positive impact on the users’ emotional expressions in the current week.*

**Hypothesis** **2** **(H2).** *The emotions received by users in the previous week have a positive impact on the users’ emotional expressions in the current week.*

**Figure 1:**
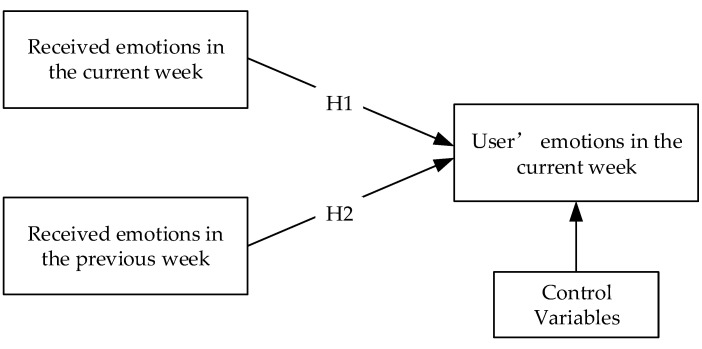
Research model.

Figure 1 shows the research model of our study. The user’s *EV_it_* (in the current week) was calculated as the dependent variable. The user’s *REV_it_* (in the current week) and *REV_it−_*_1_ (in the previous week) were calculated as independent variables. In addition, the user’s *EV_it−_*_1_ (in the previous week) was included as the control variable to eliminate serial correlation in the errors and to control the user’s intrinsic and stable emotional states. Then, we obtained the temporal sequences of all variables for each user.

Generalized estimating equation (GEE) is a general statistical approach that facilitates the analysis of data collected in longitudinal measures designs [51]. GEE has been popularly applied in clinical trials and biomedical studies [52,53]. Some studies use GEE to verify the spread of depression in real social networks [2]. Therefore, the models were estimated using a GEE. An independent working correlation structure was assumed for the clusters [54].

### 2.6. Elements of Emotional Contagion

Information and emotions spread in social networks similarly to pathogens [55]. Based on the three elements of epidemic transmission [56]—infection sources, transmission routes, and susceptible population—we divided the process of emotional contagion into three elements—emotional interaction information, interactive behavior, and susceptible group—and then analyzed these elements to promote positive emotional contagion and reduce negative emotional contagion.

#### 2.6.1. Emotional Interaction Information

After verifying the extent of the emotional contagion, we wanted to determine which emotional themes and language use were more likely to cause emotional contagion. Therefore, we calculated the difference of interactive information between the week when the emotional contagion occurred and the week when the emotional contagion did not occur by all sample users.

We extracted the replies in which positive emotional contagion occurred; in other words, the response received by each user extracted when users *REV_it_ >* 0 and *EV_it_ − EV_it−_*_1_ > 0. For comparison, we extracted the replies when positive emotional contagion did not occur, that is, the replies received by each user extracted when users *REV_it_* > 0 and *EV_it_ − EV_it−_*_1_ < 0. All the replies were segmented, and the positive emotion words were extracted from HowNet’s emotional dictionary [57]. Finally, the top 100 extracted emotion words were classified and counted. After considering measures of widely recognized basic emotions [58], we grouped the positive emotional words into (1) relaxed, (2) thankful, (3) praise, (4) hopeful, (5) like, (6) happiness, and (7) respect.

Similarly, we extracted the response postings when negative emotional contagion occurred (users *REV_it_* < 0 and users *EV_it_ − EV_it−_*_1_ < 0) and did not occur (users *REV_it_* < 0 and users *EV_it_ − EV_it−_*_1_ > 0). All of the response postings were segmented, and the top 100 negative emotion words were extracted. Negative emotions include (1) disgust, (2) fear, (3) anxiety, (4) boredom, (5) guilt, (6) hopelessness, (7) sadness, and (8) anger.

In addition, we also assessed the use of pronouns, which are categorized by the use of the first person and the second person, in positive and negative interactions.

#### 2.6.2. Interactive Behavior

To facilitate the monitoring of user interaction behavior, the distribution of positive and negative interactions over 24 h was investigated.

#### 2.6.3. Characteristics of Susceptible Groups

Finally, in order to explore if different users have different susceptibility to emotional contagion, we designed and calculated the user susceptibility index as follows:

$$SI_{pos}=\frac{C_{imp}}{C_{pos}}\;\mathrm{and}\;SI_{neg}=\frac{C_{wor}}{C_{neg}}$$

where *SI_pos_* is the positive susceptibility index, *C_imp_* is the total number of weeks when users *REV_it_* > 0 and *EV_it_ − EV_it−_*_1_ > 0, *C_pos_* is the total number of weeks when users *REV_it_* > 0. *SI_neg_* is the negative susceptibility index, *C_wor_* is the total number of weeks when users *REV_it_* < 0 and *EV_it_ − EV_it_*_−1_ <0, *C_neg_* is the total number of weeks when users *REV_it_* < 0. Users with *SI_pos_* > 0.85 or *SI_neg_* > 0.85 are considered as susceptible groups, while users with probability *SI_pos_* < 0.15 or *SI_neg_* < 0.15 are considered as non-susceptible groups. This threshold has been used to divide those that are highly and scarcely susceptible to emotional contagion [59]. For example, if a user has been active in the community for 10 weeks, their *REV_it_* > 0 in 5 weeks, and in 4 of these 5 weeks, their emotions have improved, then their *SI_pos_* = 0.8. In addition, if the user’s *REV_it_* < 0 during the other 5 weeks, and in one of these 5 weeks, their emotions worsened, then their *SI_neg_* = 0.2. Then, the user is considered among both the positive susceptible group and negative non-susceptible group. 

We compared the community participation and demographic characteristics (gender) of the susceptible and non-susceptible groups. The users’ community activity and span time were defined, that is, the total number of posts and the duration of engagement with the community. The Wilcoxon rank-sum and signed-rank test [60] was adopted to determine whether there was a statistically significant difference in the distributions of activity and span time between the two groups. Pearson’s chi-square test was used to determine whether there was a statistically significant difference in gender distribution.

## 3. Results

### 3.1. Longitudinal Changes

**Figure 2:**
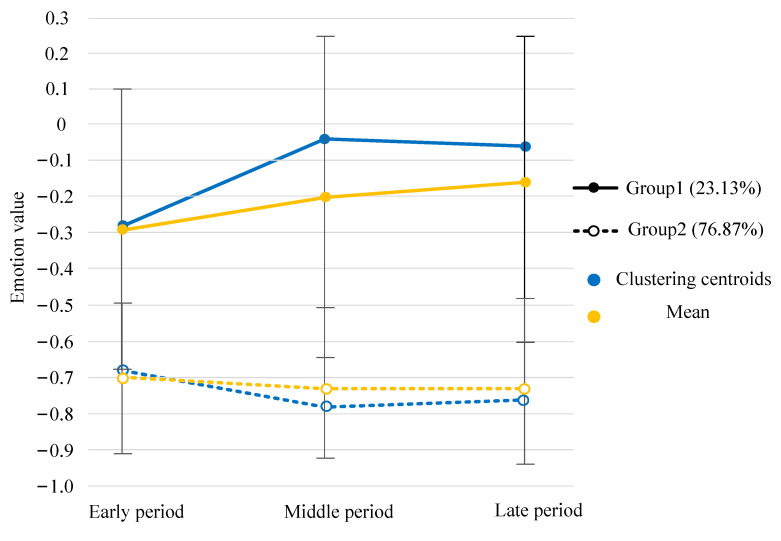
Longitudinal emotional changes for sample users in the ODC.

After obtaining the longitudinal emotional changes for each user by emotion classification and calculation, we clustered the longitudinal emotional changes for these users. The K-means clustering results show that the optimal number of clusters is 2. The clustering results and the proportion of each group are shown in Figure 2. In addition, we plotted the mean and standard deviation of EV for each group at each period in Figure 2. Group 1, with mild negative emotions, accounted for a small proportion of the sample users in the community (23.13%). This group’s posts contained fewer negative emotions in the later phase of their participation compared to the earlier period. Group 2, with more negative emotions, accounted for 76.87%. During participation in the community, the emotions of this group are relatively stable, and negative emotions tend to increase in the later period. In other words, only a small number of sample users in the community improved their emotions.

### 3.2. Emotional Contagion

The GEE regression models provide parameter estimates in the form of β-coefficients. As shown in Table 1, the results suggest that the user’s emotion in the current week is affected by the emotion received in the current week. In addition, the emotion received in the previous week has no significant impact on the user’s emotion in the current week. Therefore, it is evident that there is emotional contagion in the ODC and that it occurs on a short time scale. To check for multicollinearity, we measured the variance inflation factor for all variables. All variance inflation factors were far below the value of 2.5.

### 3.3. Elements of Emotional Contagion

#### 3.3.1. Themes of Emotional Interaction Information

**Figure 3:**
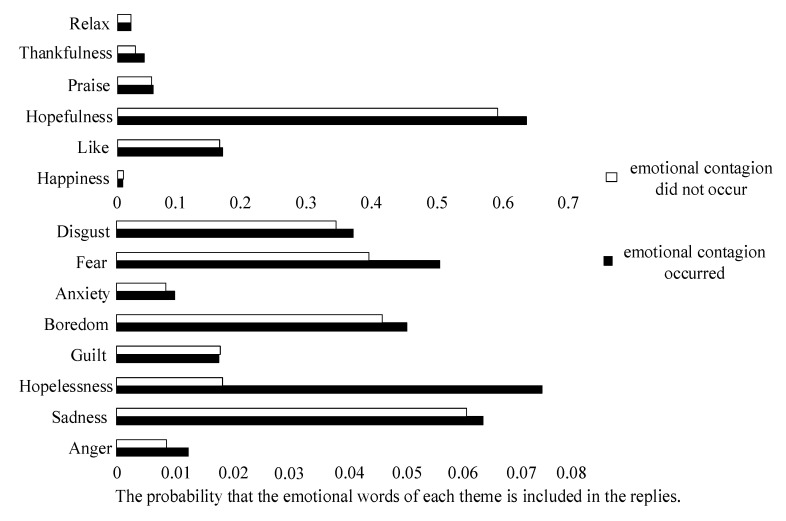
The themes of emotional interaction.

Figure 3 shows the probability of emotional interaction themes appearing in the replies. As shown, the common emotional themes in positive interactions were hopefulness, like, and praise. Replies that were more hopeful improved the recipient’s emotion. In negative interactions, users expressed more sadness, hopelessness, and fear. Among them, replies containing more hopelessness and fear were more likely to have a negative impact on the recipient. In addition, we also counted pronoun use, categorized by person; the first person was used more frequently in negative interactions, while the second person was used more frequently in positive interactions.

#### 3.3.2. Diurnal Pattern of Interaction

**Figure 4:**
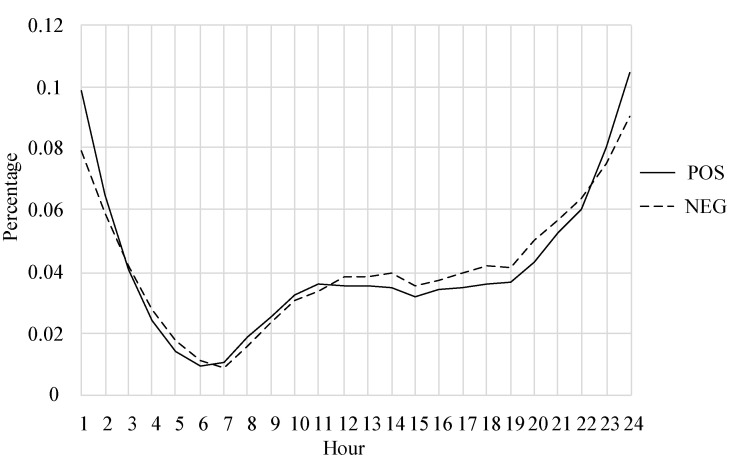
Time distribution of interaction.

Figure 4 shows the diurnal pattern of positive and negative emotional interactions of users in the community. The period from 22:00 to 02:00 was found to be the frequent time for user interaction; during this period, positive interactions were more common than negative interactions. Second, for the period from 11:00 to 22:00, user interaction was also relatively active, and negative interactions were more frequent than positive interactions. Therefore, interactions during these periods should also be considered.

#### 3.3.3. Characteristics of Susceptible Groups

**Figure 5:**
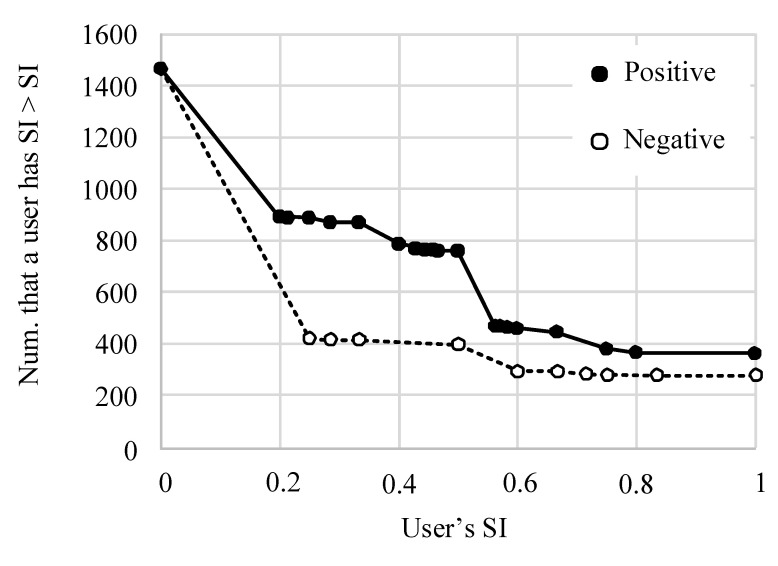
Complementary cumulative distribution of the user’s SI.

Figure 5 shows the complementary cumulative distribution of the user’s positive and negative susceptibility indices (SIs). As shown, users were more susceptible to positive emotions; that is, positive emotions were easier to spread than negative emotions.

We examined the online characteristics of susceptible and non-susceptible groups, including gender, activity, and span time. As shown in Table 2, the activity of the susceptible groups was generally higher than that of the non-susceptible groups. Users who posted and interacted frequently in the community were more likely to be affected by emotions from interactions, whether positive or negative. However, there was no significant difference in community time span between the susceptible and non-susceptible groups. In addition, the number of female users was three times that of male users. The results suggest that there was no significant difference in gender distribution between the susceptible and non-susceptible groups. The fact that no significant differences were found may be related to the small size of our sample. Using the power analysis, in order to find significant differences with some probability (power = 0.8, sig. level = 0.05), it would require expanding the data sample to at least 12 times the current size.

**Figure 6:**
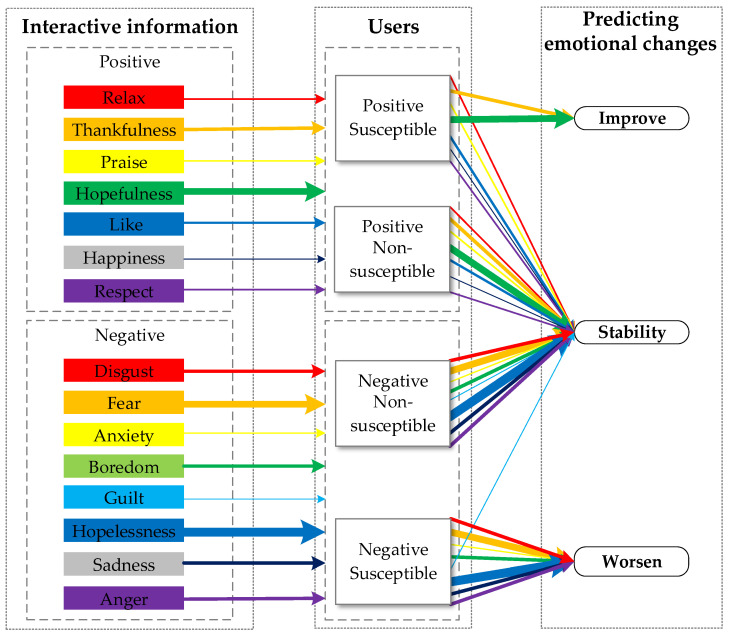
Model of users’ online behavior. Note: The thickness of the line represents the degree of influence of different emotional themes.

Finally, based on the results of this study, a model of users’ online behavior was designed to predict users’ emotional changes after interactions in ODC, as shown in Figure 6. The model describes the likelihood that users with different levels of susceptibility will improve or worsen their emotions after receiving different emotional messages. The purpose of the model is to help community managers identify individuals who are affected by others’ negative emotions to intervene and assist the identified individuals in a timely manner.

## 4. Discussion

This study examined emotional contagion in the ODC, including the three elements of emotional contagion: themes of emotional interaction, diurnal pattern of interaction, and characteristics of the susceptible group.

First, according to the emotional longitudinal change, we found that the sample users of the community were mainly divided into two groups. One was the group with mild negative emotions, which accounted for a small proportion of the sample users. This group generally showed a trend toward emotional improvement during their participation in ODC. The other group had considerable negative emotions; they comprised the main sample users, showing a slight trend toward worsening negative emotions. This emotional longitudinal change was different from results of previous studies based on online healthy communities [33,35,36] that found that online interaction can improve depression and anxiety. Our results showed that the impact of the ODC on users is complex and cannot be generalized. The frequent expression of negative emotions in online communities was associated with higher levels of depression symptoms [61,62,63]. Therefore, for the second group that had considerable negative emotions, the trend of worsening emotions may be accompanied by the aggravation of depression symptoms, which should be of concern to community managers and support groups.

Second, after understanding the emotional changes of users in ODCs, we verified that the emotions received by users in the interaction will have an impact on users’ emotions; that is, there was evidence of emotional contagion in the community. Although studies have shown that the suppression of emotional expressions backfires for depression and accepting one’s feelings, even negative ones, is the key to psychological well-being [64], online communities might be a platform that enables individuals to exchange their feelings and thoughts. However, given the massive scale of social networks such as the ODC, even small effects can have significant aggregated consequences. Frequent negative emotion exchange in ODCs will expand the emergence and spread of negative emotions, which is a potential risk. Therefore, community managers need to pay attention to negative communication and their negative impact in ODCs. At the same time, in order to promote the spread of positive emotions, the community should introduce more emotional support groups to encourage community members to actively deal with the disease. In addition, we found that emotional contagion exists on a short time scale, which requires managers to take timely and continuous response measures.

Third, through the themes of emotional interaction and use of language in the process of user interaction, we found that users pay more attention to interactive objects by using the second person in the positive emotional interaction. The common emotional themes in positive interactions were hopefulness, like, and praise. We believe that most of the positive interactions are designed to provide emotional support. Previous studies have shown that emotional support can help patients cope better with depression [53]. However, emotional support can also be divided into many categories. Compared with like and praise, emotional support with more hopefulness can have a more positive impact on users. In addition, in negative interactions, users were more likely to express their feelings by using the first person. Users expressed more sadness, hopelessness, and fear. Among them, hopelessness and fear could significantly affect the emotions of the receiving users. Previous studies have also shown that content that evokes high-arousal emotions (e.g., fear) is more likely to spread than content that evokes low-arousal emotions (e.g., sadness) [65]. Communication with despair and fear often contain a lot of discussions about death and suicide, which can convey wrong health concepts and behaviors in patients. Studies have found that the more frequently users communicate with people with suicidal ideation, the more likely that users would become suicidal [47]. Therefore, we should design a text monitoring system based on the high-frequency emotional words related to hopelessness and fear that we have collected. When such words appear in interactions, the support group can be informed to facilitate a timely intervention.

Fourth, in the diurnal pattern of user interaction, the period of frequent interaction from 22:00 to 02:00 should be focused on for monitoring, as should the afternoon and evening period of 11:00 to 22:00, in which users have relatively more negative interactions. Intervening and helping users when they are most active would be more effective. In addition, users interact frequently at night, indicating that they may have insomnia problems, so the help group should pay attention to the possibility of other complications.

Fifth, by calculating the SI, we found that positive emotions are easier to spread than negative emotions. Therefore, encouraging positive interaction among users could effectively improve the emotional state of the community. By defining and analyzing the susceptible group characteristics, we found that groups susceptible to both positive and negative emotions are more active users in the community. Such groups may be more dependent on the community. Groups susceptible to negative emotions constitute the main category of users that community managers need to pay attention to. Identifying these groups and encouraging them to communicate with positive groups could effectively prevent the spread of negative emotions.

In China, the scarcity of mental health services and resources leads to 90% of individuals with depression not being treated [45,66]. In addition, the influence of traditional culture has deepened the stigma of mental illness, encouraging depressed people to hide their depression in real life [67]. ODCs provide a platform for them to express their thoughts and communicate with each other, as well as provide the opportunity to gain health-related knowledge. However, our findings show that most users of ODCs do not improve their depression during their participation. Long-term exposure to negative emotions in the community may exacerbate levels of depression symptoms. ODCs, which gather a large number of people with depression, are the best platform for implementing online interventions at low cost and should be given high priority by mental health institutions in China.

This study has some limitations that need to be addressed in future research. First, we are not aware of whether users of ODC have depression. Further research is needed to determine the depression levels of the users. In addition, additional personal information about the users, such as their degree of depression, age, or education level, may affect the level of emotional contagion. In the follow-up study, additional user information should be obtained by means of scale and questionnaire surveys, which can be added to the model variables. Finally, we only used data from a single ODC. In a follow-up study, additional ODCs from more platforms will be included to expand our study sample size and verify the research results.

Despite these shortcomings, this study provides evidence of emotional contagion in ODCs. When positive emotional contagion occurred, the replies contained more hopefulness, and when negative emotional contagion occurred, the replies contained more hopelessness and fear. Compared with negative emotions, positive emotions are easier to spread, and people with higher activity were more susceptible. In addition, night time was an active period for user interaction. These results can potentially help community managers and support groups in taking measures to promote the spread of positive emotions and reduce the incidence of negative emotions, responding to signs of distress in a timely manner.

## Figures and Tables

**Table 1:** Results of generalized estimation equation for emotional contagion.

	Dependent Variable: Current User’s EV		
	Co-Eff	S.E.	*p*-Value
Current EV received	0.031	0.0101	0.001
Previous EV received	−0.017	0.0098	0.087
Previous user’s EV	0.500	0.0250	0.000
Constant	−0.248	0.0152	0.000

Note: EV—emotional value.

**Table 2:** Characteristics of the susceptible population.

	Positive Emotional Contagion			Negative Emotional Contagion		
	Susceptible Groups: 320	Non-Susceptible Groups: 603	*p*	Susceptible Groups: 278	Non-Susceptible Groups: 1045	*p*
**Gender**			0.435			0.466
No. of males, n (%),	73, 22.81	153, 25.37		70, 25.18	239, 22.87	
No. of females, n (%)	247, 77.19	450, 74.63		208, 74.82	806, 77.13	
**Activity**			<0.01			<0.01
Mean	157.55	131.91		221	154	
Median	97	79		117	93	
**Loyalty**			0.73			0.48
Mean	296.04	306.79		332	291	
Median	205	202		212	205	

## Data Availability

Publicly available datasets were analyzed in this study. This data can be found here: [https://m.weibo.cn/1648007681/3424883176420210] (accessed on 19 November 2021).

## References

[1] Fowler J.H., Christakis N.A. (2008). Dynamic spread of happiness in a large social network: Longitudinal analysis over 20 years in the Framingham Heart Study. BMJ.

[2] Rosenquist J.N., Fowler J.H., Christakis N.A. (2011). Social Network Determinants of Depression. Mol. Psychiatry.

[3] Cacioppo J.T., Fowler J.H., Christakis N.A. (2009). Alone in the crowd: The structure and spread of loneliness in a large social network. J. Personal. Soc. Psychol..

[4] Hatfield E., Cacioppo J.T., Rapson R.L. (1994). Emotional contagion: Cambridge studies in emotion and social interaction. Cambridge, UK: Cambridge University Press. errors-in-variables regression model when the variances of the measurement errors vary between the observations. Stat. Med..

[5] Larson R.W., Almeida D.M. (1999). Emotional transmission in the daily lives of families: A new paradigm for studying family process. J. Marriage Fam..

[6] Barsade S.G. (2002). The ripple effect: Emotional contagion and its influence on group behavior. Adm. Sci. Q..

[7] Howes M.J., Hokanson J.E., Loewenstein D.A. (1985). Induction of depressive affect after prolonged exposure to a mildly depressed individual. J. Personal. Soc. Psychol..

[8] Bono J.E., Ilies R. (2006). Charisma, positive emotions and mood contagion. Leadersh. Q..

[9] Van Kleef G.A., De Dreu C.K., Manstead A.S. (2004). The interpersonal effects of anger and happiness in negotiations. J. Personal. Soc. Psychol..

[10] Fowler J.H., Christakis N.A. (2010). Cooperative behavior cascades in human social networks. Proc. Natl. Acad. Sci. USA.

[11] Gill A.J., Gergle D., French R.M., Oberlander J. Emotion rating from short blog texts. Proceedings of the SIGCHI Conference on Human Factors in Computing Systems.

[12] Choi S., Kim E.M. (2020). Between Instagram browsing and subjective well-being: Social comparison or emotional contagion?. Media Psychol..

[13] Counts M.D.C.S., Gamon M. Not all moods are created equal! exploring human emotional states in social media. Proceedings of the International AAAI Conference on Web and Social Media (ICWSM).

[14] Coviello L., Fowler J.H., Franceschetti M. (2014). Words on the web: Noninvasive detection of emotional contagion in online social networks. Proc. IEEE.

[15] Coviello L., Sohn Y., Kramer A.D., Marlow C., Franceschetti M., Christakis N.A., Fowler J.H. (2014). Detecting emotional contagion in massive social networks. PLoS ONE.

[16] Garcia D., Garas A., Schweitzer F. (2012). Positive words carry less information than negative words. EPJ Data Sci..

[17] Goldenberg A., and Gross J.J. (2020). Digital emotion contagion. Trends Cogn. Sci..

[18] Golder S.A., Macy M.W. (2011). Diurnal and seasonal mood vary with work, sleep, and daylength across diverse cultures. Science.

[19] Hancock J.T., Gee K., Ciaccio K., Lin J.M.H. I’m sad you’re sad: Emotional contagion in CMC. Proceedings of the 2008 ACM Conference on Computer Supported Cooperative Work.

[20] Harris R.B., Paradice D. (2007). An investigation of the computer-mediated communication of emotions. J. Appl. Sci. Res..

[21] Mei Q., Ling X., Wondra M., Su H., Zhai C. Topic sentiment mixture: Modeling facets and opinions in weblogs. Proceedings of the 16th international conference on World Wide Web.

[22] Kramer A.D. The spread of emotion via Facebook. Proceedings of the SIGCHI Conference on Human Factors in Computing Systems.

[23] Kramer A.D., Guillory J.E., Hancock J.T. (2014). Experimental evidence of massive-scale emotional contagion through social networks. Proc. Natl. Acad. Sci. USA.

[24] Dang-Xuan L., Stieglitz S. Impact and Diffusion of Sentiment in Political Communication-An Empirical Analysis of Political Weblogs. Proceedings of the ICWSM.

[25] Wang J., Wei L. (2020). Fear and hope, bitter and sweet: Emotion sharing of cancer community on twitter. Soc. Media Soc..

[26] Brady W.J., Crockett M.J. (2019). How Effective Is Online Outrage?. Trends Cogn. Sci..

[27] Ba S., Wang L. (2013). Digital health communities: The effect of their motivation mechanisms. Decis. Support Syst..

[28] Fox S. (2011). Peer-to-Peer Healthcare. Many People–Especially Those Living with Chronic or Rare Diseases–Use Online Connections to Supplement Professional Medical Advice [Pew Internet & American Life Project].

[29] Bargh J.A., McKenna K.Y., Fitzsimons G.M. (2002). Can you see the real me? Activation and expression of the “true self” on the Internet. J. Soc. Issues.

[30] Kallinikos J., Tempini N. (2014). Patient data as medical facts: Social media practices as a foundation for medical knowledge creation. Inf. Syst. Res..

[31] Bartlett Y.K., Coulson N.S. (2011). An investigation into the empowerment effects of using online support groups and how this affects health professional/patient communication. Patient Educ. Couns..

[32] Griffiths K.M., Calear A.L., Banfield M. (2009). Systematic review on Internet Support Groups (ISGs) and depression (1): Do ISGs reduce depressive symptoms?. J. Med. Internet Res..

[33] Setoyama Y., Yamazaki Y., Namayama K. (2011). Benefits of peer support in online Japanese breast cancer communities: Differences between lurkers and posters. J. Med. Internet Res..

[34] van Uden-Kraan C.F., Drossaert C.H., Taal E., Shaw B.R., Seydel E.R., van de Laar M.A. (2008). Empowering processes and outcomes of participation in online support groups for patients with breast cancer, arthritis, or fibromyalgia. Qual. Health Res..

[35] van Uden-Kraan C.F., Drossaert C.H., Taal E., Seydel E.R., van de Laar M.A. (2009). Participation in online patient support groups endorses patients’ empowerment. Patient Educ. Couns..

[36] Høybye M.T., Dalton S.O., Deltour I., Bidstrup P.E., Frederiksen K., Johansen C. (2010). Effect of Internet peer-support groups on psychosocial adjustment to cancer: A randomised study. Br. J. Cancer.

[37] Shaw B.R., Hawkins R., McTavish F., Pingree S., Gustafson D.H. (2006). Effects of insightful disclosure within computer mediated support groups on women with breast cancer. Health Commun..

[38] Park A., Conway M. (2017). Longitudinal changes in psychological states in online health community members: Understanding the long-term effects of participating in an online depression community. J. Med. Internet Res..

[39] World Health Organization (2017). Depression and Other Common Mental Disorders: Global Health Estimates (No. WHO/MSD/MER/2017.2).

[40] Barney L.J., Griffiths K.M., Banfield M.A. (2011). Explicit and implicit information needs of people with depression: A qualitative investigation of problems reported on an online depression support forum. BMC Psychiatry.

[41] De Choudhury M., De S. Mental health discourse on reddit: Self-disclosure, social support, and anonymity. Proceedings of the Eighth International AAAI Conference on Weblogs and Social Media.

[42] Thompson R.J., Mata J., Jaeggi S.M., Buschkuehl M., Jonides J., Gotlib I.H. (2012). The everyday emotional experience of adults with major depressive disorder: Examining emotional instability, inertia, and reactivity. J. Abnorm. Psychol..

[43] Hatfield E., Cacioppo J.T., Rapson R.L. (1993). Emotional contagion. Curr. Dir. Psychol. Sci..

[44] Sina Weibo Data Center (2013). Weibo User Development Report.

[45] Tang J., Yu G., Yao X. (2020). A comparative study of online depression communities in china. Int. J. Environ. Res. Public Health.

[46] Wang Z., Yu G., Tian X., Tang J., Yan X. (2018). A study of users with suicidal ideation on sina weibo. Telemed. J. E Health.

[47] Wang Z., Yu G., Tian X. (2018). Exploring behavior of people with suicidal ideation in a Chinese online suicidal community. Int. J. Environ. Res. Public Health..

[48] Benton A., Coppersmith G., Dredze M. Ethical Research Protocols for Social Media Health Research. Proceedings of the First ACL Workshop on Ethics in Natural Language Processing.

[49] Devlin J., Chang M.W., Lee K., Toutanova K. (2018). Bert: Pre-training of deep bidirectional transformers for language understanding. arXiv.

[50] Charrad M., Ghazzali N., Boiteau V., Niknafs A. (2014). Nbclust: An r package for determining the relevant number of clusters in a data set. J. Statal Softw..

[51] Liang K.Y., Zeger S.L. (1986). Longitudinal data analysis using generalized linear models. Biometrika.

[52] Ballinger G.A. (2004). Using generalized estimating equations for longitudinal data analysis. Organ. Res. Methods.

[53] Wang M. (2014). Generalized estimating equations in longitudinal data analysis: A review and recent developments. Adv. Stat..

[54] Schildcrout J.S., Heagerty P.J. (2005). Regression analysis of longitudinal binary data with time-dependent environmental covariates: Bias and efficiency. Biostatistics.

[55] Hodas N.O., Lerman K. (2014). The simple rules of social contagion. Sci. Rep..

[56] Hill A.L., Rand D.G., Nowak M.A., Christakis N.A. (2010). Emotions as infectious diseases in a large social network: The SISa model. Proc. R. Soc. B Biol. Sci..

[57] Yan J., Bracewell D.B., Ren F., Kuroiwa S. (2008). The creation of a Chinese emotion ontology based on HowNet. Eng. Lett..

[58] Ekman P. (1993). Facial expression and emotion. Am. Psychol..

[59] Ferrara E., Yang Z. (2015). Measuring emotional contagion in social media. PLoS ONE.

[60] Woolson R.F. (2007). Wilcoxon signed-rank test. Wiley Encycl. Clin. Trials.

[61] Cavazos-Rehg P.A., Krauss M.J., Sowles S., Connolly S., Rosas C., Bharadwaj M., Bierut L.J. (2016). A content analysis of depression-related tweets. Comput. Hum. Behav..

[62] De Choudhury M., Counts S., Horvitz E. Social media as a measurement tool of depression in populations. Proceedings of the 5th Annual ACM Web Science Conference.

[63] Settanni M., Marengo D. (2015). Sharing feelings online: Studying emotional well-being via automated text analysis of Facebook posts. Front. Psychol..

[64] Gross J. (1998). Antecedent- and response-focused emotion regulation: Divergent consequences for experience, expression, and physiology. J. Personal. Soc. Psychol..

[65] Berger J., Milkman K.L. (2012). What makes online content viral?. J. Mark. Res..

[66] Phillips M.R., Zhang J., Shi Q., Song Z., Ding Z., Pang S., Li X., Zhang Y., Wang Z. (2009). Prevalence, treatment, and associated disability of mental disorders in four provinces in China during 2001–2005: An epidemiological survey. Lancet.

[67] Yang L.H., Kleinman A. (2008). Face and the embodiment of stigma in china: The cases of schizophrenia and aids. Soc. Sci. Med..

